# A novel paradigm for assessing olfactory working memory capacity in mice

**DOI:** 10.1038/s41398-020-01120-w

**Published:** 2020-12-15

**Authors:** Geng-Di Huang, Li-Xin Jiang, Feng Su, Hua-Li Wang, Chen Zhang, Xin Yu

**Affiliations:** 1 Peking University Sixth Hospital, 100191 Beijing, China; 2 Peking University Institute of Mental Health, 100191 Beijing, China; 3 NHC Key Laboratory of Mental Health (Peking University), 100191 Beijing, China; 4 National Clinical Research Center for Mental Disorders (Peking University Sixth Hospital), 100191 Beijing, China; 5 Beijing Municipal Key Laboratory for Translational Research on Diagnosis and Treatment of Dementia, 100191 Beijing, China; 6 Peking-Tsinghua Center for Life Sciences, Academy for Advanced Interdisciplinary Studies, Peking University, 100871 Beijing, China; 7 School of Basic Medical Sciences, Beijing Key Laboratory of Neural Regeneration and Repair, Advanced Innovation Center for Human Brain Protection, Capital Medical University, 100069 Beijing, China

**Keywords:** Psychiatric disorders, Neuroscience

## Abstract

A decline in working memory (WM) capacity is suggested to be one of the earliest symptoms observed in Alzheimer’s disease (AD). Although WM capacity is widely studied in healthy subjects and neuropsychiatric patients, few tasks are developed to measure this variation in rodents. The present study describes a novel olfactory working memory capacity (OWMC) task, which assesses the ability of mice to remember multiple odours. The task was divided into five phases: context adaptation, digging training, rule-learning for non-matching to a single-sample odour (NMSS), rule-learning for non-matching to multiple sample odours (NMMS) and capacity testing. During the capacity-testing phase, the WM capacity (number of odours that the mice could remember) remained stable (average capacity ranged from 6.11 to 7.00) across different testing sessions in C57 mice. As the memory load increased, the average errors of each capacity level increased and the percent correct gradually declined to chance level, which suggested a limited OWMC in C57 mice. Then, we assessed the OWMC of 5 × FAD transgenic mice, an animal model of AD. We found that the performance displayed no significant differences between young adult (3-month-old) 5 × FAD mice and wild-type (WT) mice during the NMSS phase and NMMS phase; however, during the capacity test with increasing load, we found that the OWMC of young adult 5 × FAD mice was significantly decreased compared with WT mice, and the average error was significantly increased while the percent correct was significantly reduced, which indicated an impairment of WM capacity at the early stage of AD in the 5 × FAD mice model. Finally, we found that FOS protein levels in the medial prefrontal cortex and entorhinal cortex after the capacity test were significantly lower in 5 × FAD than WT mice. In conclusion, we developed a novel paradigm to assess the capacity of olfactory WM in mice, and we found that OWMC was impaired in the early stage of AD.

## Introduction

Alzheimer’s disease (AD), the most common cause of dementia in elderly individuals, is characterised by progressive loss of cognitive abilities^1–3^. Most research has focused on understanding the relationship between memory impairments and the pathological hallmarks of the disease, namely, the presence of amyloid-beta (Aβ) accumulation in amyloid plaques, tau aggregation in neurofibrillary tangles and brain atrophy caused by loss of neurons and synapses^4,5^. A reliable and objective means of early detection would not only allow incipient AD to be identified before clinical diagnostic criteria are fulfilled but also have an important role in potential early intervention^6–8^. Deficits in working memory (WM) have a central role in normal neurocognitive ageing and the rapid cognitive deterioration associated with dementias such as Alzheimer’s disease^9,10^. There has been growing interest in how WM is affected in the early stages of AD. Deficits in WM in AD are thought to contribute to a range of significant problems, such as difficulties in dividing attention and manipulating remembered information^11^. Compared with health controls, AD patients are more easily overloaded by information and show more rapid performance decline with increased task demands, suggesting an impairment of WM^12^. Moreover, presymptomatic individuals have difficulty recalling items, indicating a deficit in WM in the early stages of AD^8^. Thus, uncovering the mechanism underlying WM may aid in developing measures to prevent memory impairment in AD^13^.

WM, the ability to maintain and process information over a period of seconds to organise goal-directed behaviour, is generally viewed as short-lived and affected by the delay interval^14,15^. The capacity of WM is limited, and a restricted amount of information or number of items is kept in WM at once. Miller^16^ noted that the capacity of short-term memory in humans is 7 ± 2, while Cowan^17^ claimed that the capacity limit of WM averages around the ‘magical number 4’ chunks; thus, the absolute limits remain controversial. A number of procedures have been used to measure WM in rodents, including the radial arm maze, the WM version of the Morris swim task, and a variety of delayed match- and non-match-to-sample tasks^18^. However, it should be noted that the memory component assessed in these procedures does not include memory capacity, which is often measured in human cognitive tasks. Recently, the odour span task (OST) has been increasingly used as a rodent procedure to explore WM capacity^19–26^. In this procedure, rodents are trained to apply a non-match-to-sample odour rule to identify the novel odour among several odours, which has not yet been presented in a previous trial. Thus, the number of odour stimuli to remember increases during the session, and the number of consecutive correct responses (span length) and percent correct are used to define the WM capacity^19–21^. The OST has been performed across species such as mice^22,27,28^, rats^19,25,26^ and humans^29,30^. Collectively considering the evidence from OST studies, there are two differences between OST and classic human traditional span tasks (i.e. digital span). First, in the OST design, odours that are presented early in the series appear again and again in subsequent trials; thus, mice may remember certain types of odours repeatedly. In contrast, classic WM capacity procedures for humans typically require serial recall of the to-be-remembered items, and items presented during the trial are only relevant for controlling behaviour during a single trial^31,32^. Second, the performance of subjects has revealed a high accuracy and appears to remain well above chance with a high load in OST^19,22,30^, while it has shown a significant decline with a high load in human research^33,34^. In addition, it failed to define WM capacity based on programming sessions with 36, 48 or 72 stimuli in rats that had previously received extensive OST training^35,36^, although whether capacity limits of WM could be defined in mice and humans remains unknown.

In this study, inspired by the OST task, we first developed a novel olfactory WM capacity paradigm in which we introduced a measure for the capacity of WM of trial-specific information, and then we assessed the performance of mice from a low to a high load of memory information in the paradigm. We also explored OWMC in young adult (3-month-old) 5 × FAD mice to investigate whether OWMC was impaired in the early stage of AD, and we assessed how the activation of brain regions changed when they performed the OWMC.

## Materials and methods

### Subjects

Wild-type C57BL/6 mice (males, aged 3 months, *n* = 9) were purchased from Vital River Laboratories (Beijing, China). The 5 × FAD transgenic mice (males, aged 2–3 months) were purchased from the Jackson Laboratory (Bar Harbor, ME, USA, strain no. 008730). We used heterozygous 5 × FAD (B6SJL-Tg (APP K670N/M671L + I716V + V717I and PS1 M146L + L286V)) mice on a C57BL6/SJ1 hybrid background with wild-type (WT) littermates as controls (males, aged 3 months, *n* = 10). The mice used in the experiment were the progeny of male hemizygous C57BL/6J × SJL/J FN 5 × FAD and female WT C57BL/6J × SJL/J F1 mice bred in our laboratory. The mice were kept in a temperature- and humidity-controlled environment (22 ± 2 °C, 50 ± 10%) with ad libitum access to food (SPF grade for genetically modified mice) and water under a reverse 12-h/12-h light/dark cycle with lights on at 19:00 h before the experiment. The behavioural experiments were conducted during the dark phase of the cycle. Mice were maintained at 85% of their free-feeding weight and had free access to water during training and testing for the behavioural experiments. To prevent variations in the degree of food restriction, mice were housed individually each in a cage with dimensions of 32.5 cm long × 21 cm wide × 18 cm high. Each mouse was genotyped twice to ensure the correct genetic identification. All animal studies were conducted in accordance with the Guide for the Care and Use of Laboratory Animals (8th edition) and approved by the Institutional Animal Care and Use Committee of Peking University. We further adopted the recommendations of the gold standard publication checklist (GSPC)^37^.

### Behavioural apparatus and materials

The training and test phases of the OWMC task were conducted in the same room. Briefly, context adaptation, digging training and NMSS rule-learning took place in a training cage (a clear Perspex cage, 46 cm long × 23 cm wide × 19 cm high). The training cage consisted of two chambers separated by a manual guillotine door (23 cm wide × 19 cm high). NMMS rule-learning and the capacity test phase took place on a square Perspex platform (61 cm × 61 cm) connected to a waiting zone (15 cm long × 15 cm wide × 19 cm high) and a sample zone (60 cm long × 10 cm wide × 19 cm high). The choice zone, waiting zone and sample zone rested on a wooden table 76 cm above the floor, and the three zones were separated by manual guillotine doors (14.5 cm wide × 19 cm high). Platform locations were numbered around the perimeter from 1 to 24, with number 1, 7, 13 and 19 positioned at the corners. The locations in the sample zone were numbered from 1 to 12 along with the chamber, with the locations equally spaced. The bowls containing the odours (McCormick products) could be placed at any of the above positions during training and testing. The 20 odours used were as follows: dill, cinnamon, chilli, thyme, onion, rosemary, cumin, allspice, clove, almond, mint, matcha, basil, curry, ginger, caraway, coffee, celery, white pepper, spinach^22,38^. The odours we used were referred to in Young’s study, the previous study in our lab^28^, and other studies, such as episodic odour memory of mice^38^ and other odour span task studies of rats^23,25,36,39^. Each odour bowl contained a mixture of 7 g sawdust, 1 g cheese powder and 0.5 g of the corresponding test odour. Each bowl was 5.5 cm in diameter, 3.5 cm high and marked with a number to identify the odour inside. To prevent the animals from moving the odour bowls, we placed a strip of Velcro on the bottom of each bowl; the complementary Velcro dots were placed on the training cages, sample zone and platform, allowing the bowls to be fixed in place.

### Behavioural procedure

**Fig. 1:**
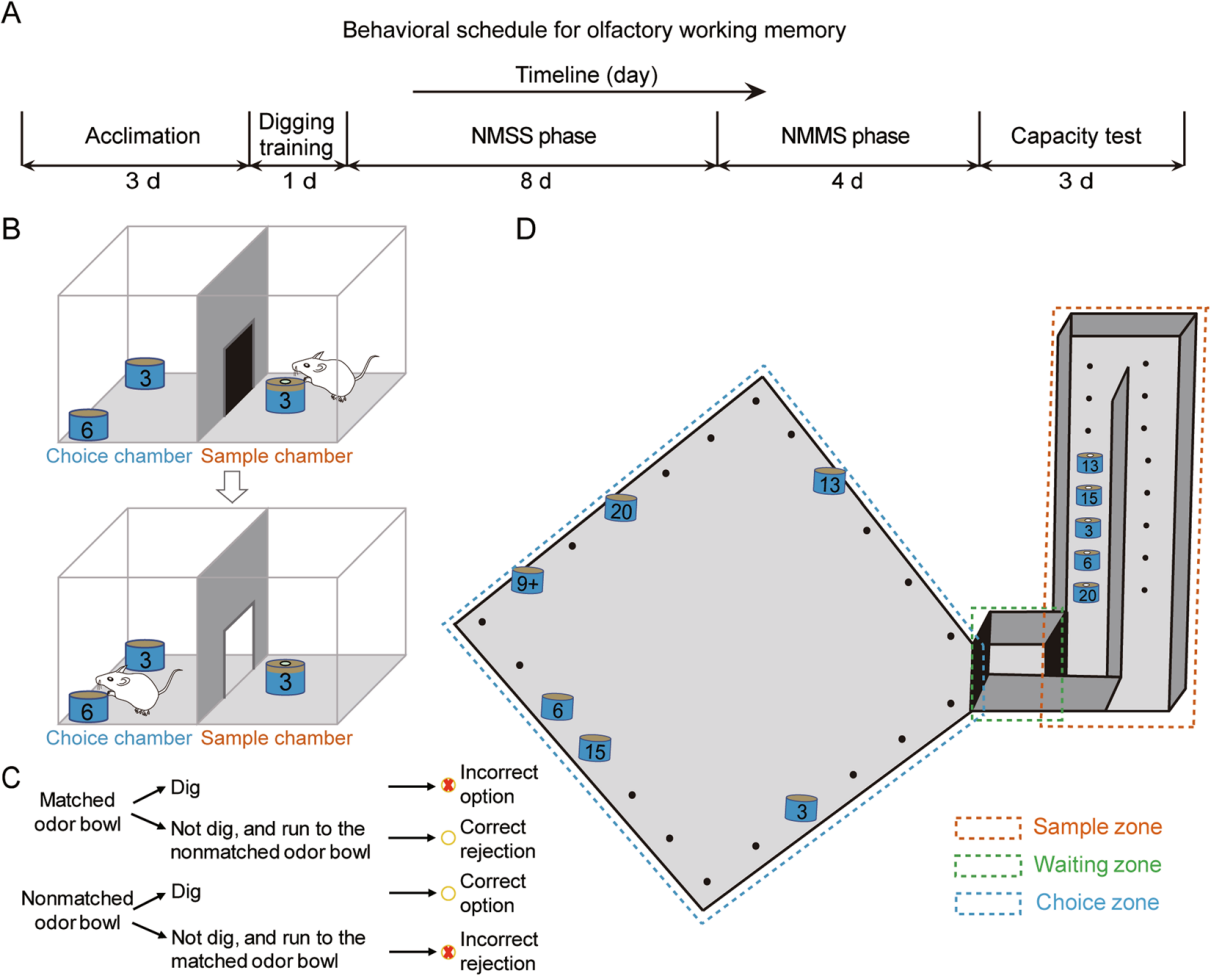
Schematic illustration of the olfactory working memory capacity task. **A** Timeline of the novel task. **B** The non-matching-to-single-sample (NMSS) learning phase of the task. During the NMSS phase, each mouse was presented with two stimuli (sample odour stimulus and novel odour stimulus) per trial and trained to dig for pellets in the scented bowls with novel odours. In each trial, mice were initially placed in the sample chamber and allowed to explore and detect the sample odour. Then, the mice were allowed to enter the choice chamber and choose between the sample odour bowl and the novel odour bowl. **C** Different types of responses in the NMSS phase. **D** The non-matching-to-multiple-sample (NMMS) learning phase and capacity-testing phase of the task. During the NMMS phase, the mice were placed in the sample zone to detect the sample stimulus, allowed to enter the waiting zone for 5 s, and then allowed to go through the door to choose between the sample odour bowl and novel odour bowl on the platform. Once the mice performed reliably in the NMMS rule-learning phases, they underwent a WM capacity test. The procedure was similar to that of the NMMS rule-learning phase, except that the number of sample odours that the mice were required to remember was gradually increased until two consecutive incorrect responses were made.

The OWMC procedure was designed based on the previous studies^22^. The task was divided into 5 phases: context adaptation, digging training, NMSS rule-learning, NMMS rule-learning and capacity testing (Fig. 1A).

#### Context adaptation

Mice were first habituated to the training cage over 3 days for 10 min/day, during which the centre door was open, allowing the mice to explore the two chambers freely.

#### Digging training

After habituation, mice were trained to dig in a bowl of unscented sawdust for a single cheese pellet^40–42^ (0.05 g) for 6 trials/day. The bowl was placed in one chamber of the training cage, and the mice were placed in another chamber, from which they were allowed to move through the centre door, dig in the sawdust and consume the cheese pellet. In the first three trials of the dig-learning phase, the cheese pellets were half buried in the sawdust, and in the remaining three trials, the pellets were buried under ~1.0 cm of sawdust with equally often locations.

#### NMSS rule-learning

After habituation, the mice received 8–12 sessions of the NMSS learning phase. The mice were trained to dig for pellets in the scented bowls with novel odours (Fig. 1B). To prevent the scented sawdust from adhering to the fur of the mice as they tipped the bowl over, a lid with a hole (1.5 cm in diameter) was placed on the sample odour bowl. The sample odour bowl was presented in one chamber of the training cage (designated the sample chamber), and a bowl with the same (match) odour and another bowl with a novel (non-matching) odour were presented on another chamber (designated the choice chamber). A cheese pellet was buried only in the novel odour bowl. In each trial, mice were initially placed in the sample chamber and allowed to explore and detect the sample odour. We considered a mouse to have investigated the odour once it had placed its nose through the hole in the cover. Then, the door between the sample chamber and the choice chamber was opened, and mice were allowed to go to the choice chamber to investigate the two bowls. Once the mice entered the choice chamber, the door was closed. If a mouse responded to the novel odour bowl and retrieved the cheese pellets, it was removed to its home cage to await the next trial. If not, the mouse was briefly returned to its home cage while the locations of the two bowls in the choice chamber were pseudorandomly reassigned, and the trial was repeated^43,44^, ensuring the pellets were retrieved only on novel odours and incorrect responses were not rewarded. After each trial, the apparatus was cleaned with disposable paper towels using 75% ethanol to reduce residual odour. We used a rigorous definition of ‘response’ in which the mouse had its forepaws or snout in physical touch with the sawdust plus digging motions, so we could gain unambiguous response results. The inter-trial interval (ITI) was 60 s during the NMSS training. In each session, 20 different odours were randomly divided into 10 pairs of sample odours and novel odours, which were assigned to the 10 trials of the session, exposing each mouse to 20 different odours, and in each trial, the locations of the two odours bowls were randomly distributed. This NMSS training was repeated for a minimum of 8 sessions, ensuring that the performance rate of each mouse reached at least 80% criteria^19,45,46^. The mice then progressed to the serial NMMS training. If a mouse missed the deadline, it was trained for 4 more sessions. Mice that failed to meet 80% criteria by session 12 were excluded from the study.

Inspired by previous studies^47,48^ and the theory of signal detection^49^, we introduced the parameters of correct option, incorrect rejection, correct rejection, incorrect option and omission to precisely analyse behaviours during the training. The behaviour results (correct option, incorrect rejection, correct rejection and incorrect option) depend on the response to the odour encountered first in each trial. When the mouse gets to the non-matching odour first (trials of encountering non-matching odour first), its responses are defined as incorrect rejection (the mouse does not dig, simultaneously turns around to the opposite matching odour bowl, and digs, then will be immediately removed) or correct option (the mouse digs into the non-matching odour bowl, then will be allowed to consume reward food). When the mouse matches the odour first (trials of encountering matching odour first), its responses are defined as correct rejection (the mouse does not dig, simultaneously turns around to the opposite non-matching odour bowl and digs, then will be allowed to consume reward food) or incorrect option (the mouse digs into the matching odour bowls, then will be immediately removed). If the mouse did not give any response within the limited time, then the omission result was obtained. The performance correct rate, correct option rate, incorrect option rate and correct rejection rate of each session were defined as follows (Fig. 1C)^48^:

Performance correct rate = (No. of correct option trials + no. of correct rejection trials)/total number of trials.

Correct option rate = No. of correct option trials / (no. of correct option trials + no. of incorrect rejection trials).

Incorrect option rate = No. of incorrect option trials / (no. of incorrect option trials + no. of correct rejection trials).

Correct rejection rate = No. of correct rejection trials / (no. of incorrect option trials + no. of correct rejection trials).

Omission = No response in 5 min.

#### Probe test

To verify that the mice were using the scent of the sawdust but not the scent of the cheese pellets to solve the task, two probe sessions were conducted on two consecutive days. The first probe was the ‘unbaited session’, in which no cheese pellet was available in the correct bowl, and a pellet was dropped into the cup only after the animal made its choice by digging in the correct bowl. The second probe was the ‘baited session’, in which cheese pellets were present in both the sample and novel odour bowls, and a mouse was allowed to consume the pellet only after the animal had made its choice by digging in the correct bowl. The probing trials were semi-randomly distributed among normal trials on each test day.

#### NMMS rule-learning

After meeting the criteria for successful NMSS rule-learning, animals were introduced to learn the NMMS rules for 4–8 sessions (Fig. 1D). In the first trial (non-matching to 1 sample odour) of each NMMS learning session, the mice were placed in the sample zone to detect the sample stimulus and then allowed to enter the waiting zone. After staying in the waiting zone for 5 s, the mice were allowed to go through the door to choose between a sample odour bowl and a novel odour bowl on the choice zone. A sample odour bowl with a lid was placed in the sample zone, while a sample odour bowl and a novel odour bowl were placed in randomly assigned locations on the choice zone. As soon as the mouse would dig in either bowl, the timer was stopped; if a mouse made the correct choice (digging into the novel odour bowl), then it was given time to consume the food reward, after which it was returned to the home cage until the next trial. Otherwise, if a mouse made the incorrect choice (digging into the sample odour bowls), then the locations of the sample and novel odour bowls in the choice zone were randomly reassigned, and the trial was repeated until the mice made the correct choice. In the second NMMS trial (non-matching to 2 sample odours), another two new sample odours were randomly chosen from the pool of odour stimuli and placed in the sample zone, and the choice odour bowls, including two sample odour bowls and a novel odour bowl, were placed on the choice zone. Only when all odours were sampled could the mice enter the waiting zone. The process was repeated for an additional 3 trials, such that there were 5 sample odour bowls in the fifth trial. At the beginning of each session, sample and choice odours for each of 5 trials were randomly selected among twenty different odours randomly assigned to 5 trials using sample and choice odours, ensuring that each animal was exposed to all twenty odours regularly over the course of training. In each trial, the locations of odour bowls in the sample zone and on the platform were randomly distributed. The ITI was set for 3 min. The NMMS learning was repeated for a minimum of 4 sessions, ensuring that each mouse completed non-matching to 2 sample odours in 2 consecutive sessions. If the mouse failed, it was trained for 4 more sessions, and mice that still failed to complete the trial of non-matching to 2 sample odours on 2 consecutive sessions during this additional training received a memory capacity score of 1.

#### Capacity testing

After the mice performed reliably in the NMMS rule-learning phases, they began to receive 3–5 sessions of WM capacity testing. The procedure was similar to that of the NMMS rule-learning phase, except that the number of sample odours that the mice needed to remember was gradually increased until 2 consecutive incorrect responses were made, and odours were chosen from odour pools in each trial. Referring to the parameters of capacity tests in humans^32^, during the test, if the mice made a correct choice in the trial, then it progressed to next capacity level trial; if the mice made the first choice incorrectly, the positions of the odour bowls were randomly reassigned, and the test was repeated. if a mouse made 2 consecutive incorrect responses in a certain level of a capacity test (capacity level *n*), the session was terminated, and the capacity of the mouse was scored as (*n* − 1). For example, if a mouse made consecutive incorrect responses at capacity level 5 (5 sample odours were presented in the trial), then the capacity of the mouse was scored as 4. Thus, the task requires animals to remember the odour stimuli encountered in the sample phase, but this information is only relevant to the current trial and will not be relevant for subsequent trials. The correct and incorrect response were recorded each time, and averaged errors and percent correct were calculated to reflect the performance accuracy of mice at each capacity level. The percentage of mice that were still successful in the OWMC task at each test level was also calculated to compare the OWMC among different genotypes. The chance level is the level that would be expected by random choice and is compared with the percent correct. As the number of sample odours(*n*) increases, the chance of a correct choice 1/(*n* + 1)% decreases, and the chance level implies the difficulty of the OWMC task.

Percentage of mice that succeeded at each capacity level = No. of mice that succeeded in the trial at each capacity level/no. of mice in the test %.

Averaged errors = No. of all errors made by mice at each capacity level/no. of mice in the test.

Percent correct = No. of corrected trials at each capacity level/(no. of corrected trials + no. of all errors made by mice)%.

### Tissue preparation

Twenty-four hours after the capacity test, 5 × FAD mice and littermates were randomly divided into four groups: WT + no OWMC task, 5 × FAD + no OWMC task, WT + OWMC task and 5 × FAD + OWMC task. All animals underwent identical experimental manipulations such as food restriction and NMSS and NMMS trainings before assignment to different groups. Mice in the task groups underwent two capacity test trials (3-sample and 6-sample trials), while mice in the no task groups entered a cage and detected sample and choice odours freely, during which the numbers and types of odours were equivalent to the OWMC task groups. The mice then entered the empty sample zone and obtained a food pellet in the empty choice zone. Ninety minutes after the behavioural assessments, the animals were deeply anaesthetised and perfused with 0.9% saline followed by a 4% buffered formalin solution, pH 7.4. The brains were collected and postfixed in the same fixative overnight at 4 °C and then transferred to 0.1 M PBS containing 30% sucrose, pH 7.4. Then, the brains were frozen on dry ice and stored at −80 °C until sectioning. The brains were cut (40 µm slices) in the coronal plane with a Leica cryostat. The slices were collected for immunohistochemistry to detect FOS protein expression, and the remaining slices were stored at −20 °C in a cryoprotectant until processing.

### Immunohistochemistry and microscopy

Immunofluorescence assays were performed to examine FOS expression in brain slices^50^. Slices were subsequently washed three times for 5 min with 0.1 M PBS to remove OCT and blocked using 5% bovine serum albumin prepared in 0.1 M PBS containing 0.3% Triton X-100 for 1 h at 37.5 °C. Slices were incubated overnight at 4 °C with the primary antibody (FOS, 1:500, #2250s, Cell Signaling Technology) diluted in blocking solution, rinsed in 0.1 M PBS to wash away the unbound antibody, and then incubated for 2 h at room temperature in species-specific secondary antibody (Alexa Fluor 488-conjugated goat anti-rabbit, 1:500, #ab150077, Abcam). The secondary antibody was removed by three wash steps for 5 min in 0.1 M PBS, and nuclei were stained with DAPI (1:1000; Sigma Chemical) for 5 min at room temperature.

For Aβ pathology detection, we observed morphologically different plaque types using a double-staining procedure with thioflavin-S and 6E10 antibodies against Aβ. Briefly, slices were subsequently washed three times for 5 min with 0.1 M PBS to remove the cryopreservative and treated with 0.05% potassium permanganate solution at room temperature for 20 min followed by a mixture of 0.2% potassium metabisulfite and 0.2% oxalic acid for 3 min. This step was followed by incubation with 0.0125% thioflavin-S solution in 40% ethanol at room temperature for 5 min in the dark. Slices were washed with 50% ethanol for 5 min to remove the excess stain, and then blocked using 5% bovine serum albumin prepared in 0.1 M PBS containing 0.3% Triton X-100 for 1 h at 37.5 °C. Slices were incubated overnight at 4 °C in the primary antibody (6E10, 1:1000, # 803004, Biolegend) diluted in blocking solution, rinsed in 0.1 M PBS to wash away any unbound antibody, and then incubated for 2 h at room temperature with species-specific secondary antibody (Alexa Fluor 633-conjugated goat anti-mouse, 1:1000, #35512, ThermoScientific). The secondary antibody was removed by three wash steps for 5 min in 0.1 M PBS, and nuclei were stained with DAPI (1:1000; Sigma Chemical) for 5 min at room temperature. Slices were viewed and images acquired at ×20 magnification using a Zeiss Axio Observer Z1 with Zen Blue 2 software.

### Quantification of neuronal numbers

The number of Fos-labelled cells was quantified in a blinded fashion as in the previous studies^50^. Images were quantified using ImageJ, and the ‘analyse particles’ function in ImageJ was used to count the number of FOS-positive cells. Scale bars are 50 µm, and each image area is 330 µm × 330 µm.

### Statistical analysis

All analyses were performed in a double-blinded manner. Our sample sizes are similar to those reported in previous publications^51,52^. The experiments were randomised using a random number generator. The results are expressed as the mean ± SEM. The Shapiro–Wilk test was used to verify the normal distribution, and Levene’s test was used to verify the homogeneity of variance. Comparisons between two groups were conducted using the unpaired Student’s *t*-test. The data were analysed using analysis of variance (ANOVA) with appropriate between- and within-subject factors for each experiment (see ‘Results’ section).

## Results

### Acquisition and performance of the OWMC task in C57 mice

**Fig. 2:**
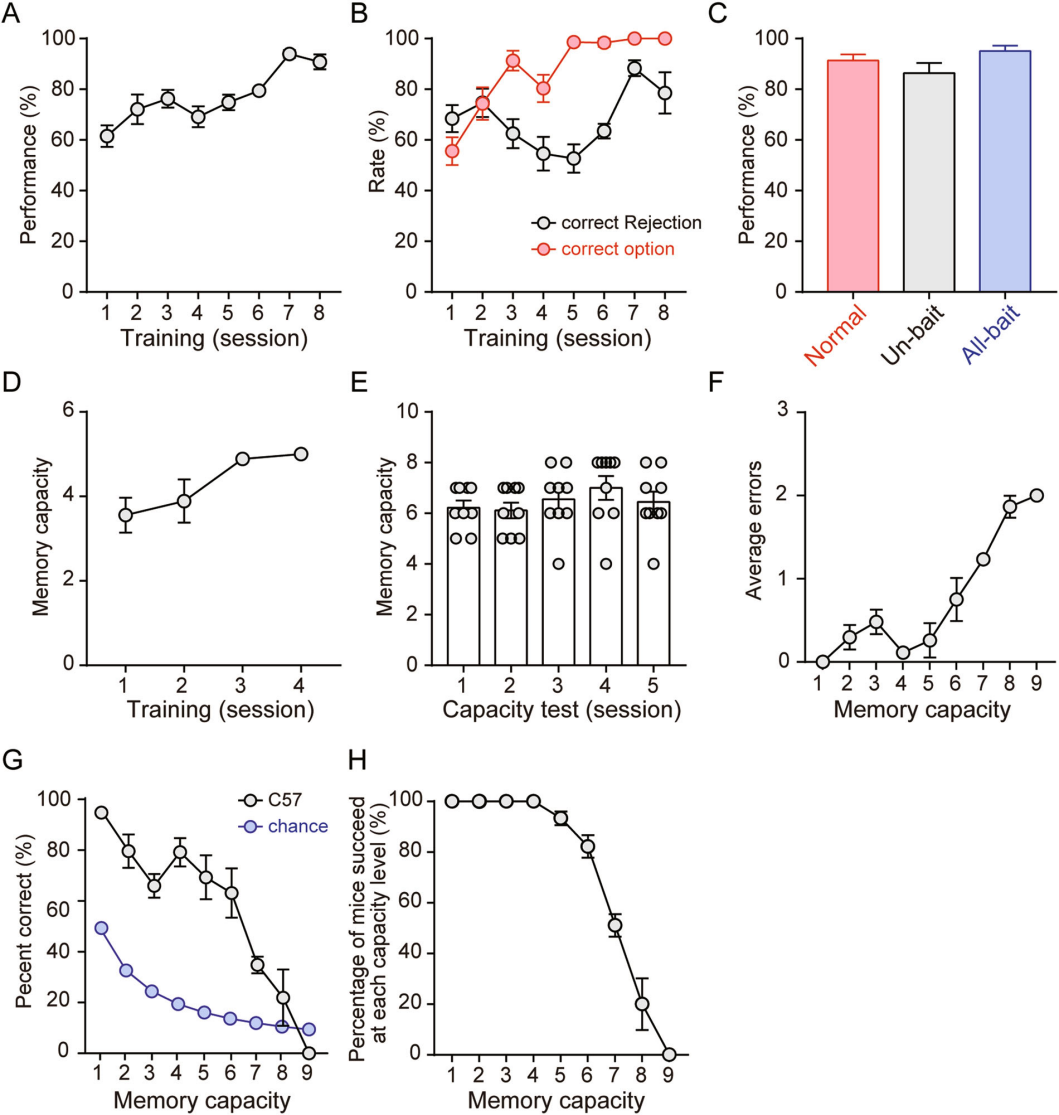
Behavioural performance of C57 mice in the olfactory working memory capacity task. **A**, **B** Performance, correct option rates and correct rejection rates in learning during the non-matching-to single-sample (NMSS) phase. **C** Performance in the probe trials. **D** The number of odours mice remembered in each trial during the non-matching-to-multiple-samples (NMMS) phase. **E** WM capacity during the test phase. **F** Average errors at each capacity level during the test phase. **G** Percent correct at each capacity level during the test phase. **H** The percentage of mice succeed at each capacity level. *n* = 9. Data are presented as the mean ± SEM.

We first used a novel paradigm, OWMC, to assess the capacity of WM in C57 mice (Fig. 2). Figure 2A shows the performance accuracy during the NMSS phase. Repeated-measures ANOVA, with a session as the within-subjects factor, was used to analyse the behavioural data during the phase. The analysis showed that the performance accuracy (*F*_7, 56_ = 8.02, *p* < 0.001; Fig. 2A), correct option rate (*F*_7, 56_ = 17.19, *p* < 0.001; Fig. 2B), and correct rejection rate (*F*_7, 56_ = 5.35, *p* < 0.001; Fig. 2B) gradually increased during the NMSS rule-learning phase, and the post hoc analysis revealed no significant differences between the last two sessions (all *p* > 0.05). In addition, we also analysed whether the appetitive/aversive value of the different natural odours could affect the performance, and we found no significant difference between the incorrect option rates of each odour when odours served as a sample odour (*χ*^2^ = 28.974, *p* > 0.05) or choice odour (*χ*^2^ = 29.633, *p* > 0.05). In addition, to exclude the possibility that the unequal ratio of ‘encountering non-matching odour first’ or ‘encountering matching odour first’ trials may affect subsequent parameter analysis, we analysed the two kinds of trials of each mouse in the NMSS phase, and we found no significant differences (*p* > 0.05) across different subjects in our study. These results indicate that the mice readily learned the rule that the new odour was an indication of food pellets.

To assess whether mice responded to the odour stimulus and not to the odour of the food pellet, unbaited and baited probe trials were conducted after the NMSS rule-learning phase. The performance accuracy rates in normal, unbaited, and baited trials were all >80%, indicating a highly reliable performance accuracy during the probe test. One-way ANOVA showed no significant differences in performance accuracy (*F*_2, 16_ = 2.40, *p* > 0.05, Fig. 2C) among the three types of trials, indicating that performance was not guided by the odour of the food pellets.

During the NMMS rule-learning phase, the number of sample odours that the mice remembered gradually increased to approximately five after four training sessions; and repeated ANOVA indicated a significant effect of sessions on memory capacity (*F*_3, 24_ = 5.46, *p* < 0.01, Fig. 2D), and the post hoc analysis revealed no significant differences between the last two sessions (*p* > 0.05).

During the five sessions of the capacity test phase, we found that the OWMC was 6.46 ± 0.15, and the OWMC each day ranged from 6.11 to 7.00. One-way ANOVA of OWMC showed no significant effect of sessions on memory capacity (*F*_4, 32_ = 1.83, *p* > 0.05, Fig. 2E), indicating that OWMC of C57 mice was stable across different sessions. Furthermore, although the OWMC varied among different individual mice, the variation in OWMC within animals across different sessions was less than 20%. In addition, one-way ANOVA showed a significant effect of sessions on averaged errors (*F*_7, 28_ = 10.14, *p* < 0.01, Fig. 2F), and the percent correct was not significantly higher than the chance level after a memory load of 8 (*p* > 0.05, Fig. 2G). As the memory load increased, the averaged errors increased while the percent correct gradually declined, and at capacity levels of 8 and 9, the percent correct showed no significant differences compared with the chance level (all *p* > 0.05, Fig. 2G). We also found that as memory load increased, along with difficulty, fewer mice succeeded in the more difficult trials (Fig. 2H). These results demonstrated that the OWMC of mice assessed by the novel procedure had a stable limit, which is similar to the memory phenomenon found in tasks of human WM.

To explore whether short-term habituation was involved in the mechanisms underlying the OWMC task, we analysed the behaviour data to determine whether the reappearance of an odour in subsequent trials might interfere with the choice. If an odour appeared as a sample odour in an early trial (e.g. capacity level 5 test) and then as the novel odour in a later trial (e.g. capacity level 6 test), it could be expected that the earlier exposure (as a sample) could affect the choice in the later trial (when the odour served as the ‘novel’ odour). We found that compared to the sub-group with no reappearance of an odour in the capacity level 6 test, the sub-group with the reappearance of an odour in the test did not show significant differences in the percent correct (*χ*^2^ = 0.113, *p* > 0.05, 65.7% vs. 61.5%).

### 5 × FAD mice show early-onset olfactory-based WM capacity deficits

**Fig. 3:**
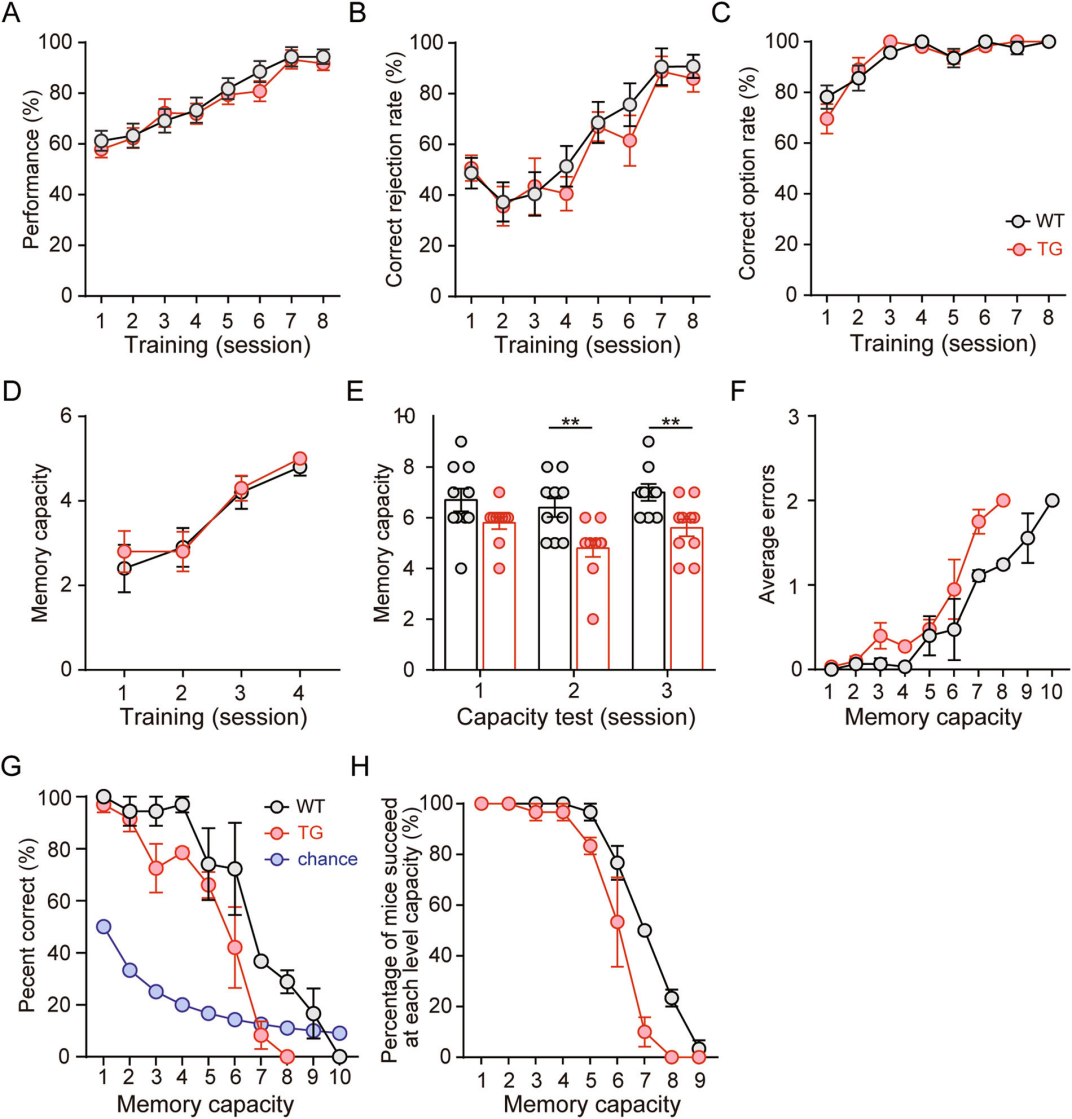
Assessing the deficit of working memory capacity of 3-month-old 5 × FAD mice using the olfactory working memory capacity task. **A**–**C** Performance, correct rejection rates and correct option rates during the non-matching-to single-sample (NMSS) phase. **D** The number of odours mice remembered in each trial during the non-matching-to-multiple-sample (NMMS) phase. **E** WM capacity during the test phase. **F** Average errors at each capacity level. **G** Percent correct at each capacity level during the test phase. **H** The percentage of mice that succeeded at each capacity level. *n* = 8–9 per experimental condition. Data are presented as the mean ± SEM. *Compared with the WT group, *p* < 0.05.

Previous human studies have suggested that WM capacity is impaired in the early stage of Alzheimer’s disease^11,53^; however, few studies have examined whether WM capacity is deficient in the early stages of AD animal models. The 5 × FAD mice contain two presenilin-1 and three amyloid precursor protein (APP) mutations: the M146L and L286V mutations in PSEN, and the Swedish (K670N/M671L), Florida (I716V), and London (V717I) mutations in APP. They develop amyloid deposition starting at ~1.5–2 months of age and neurodegeneration and cognitive deficits as early as 4–5 months of young adult age^54,55^. Thus, in Exp. 2, we used the novel rodent OWMC paradigm to assess the WM capacity in 3-month 5 × FAD mice, which showed a rare significant impairment in cognitive tasks^56,57^. Mixed ANOVAs were used to analyse the behavioural data with genotype (5 × FAD, WT) as the between-subject factor and training/test session (Session 1-n) as the within-subject factor. It was revealed that only training sessions affected performance accuracy (*F*_7, 126_ = 25.56, *p* < 0.001; Fig. 3A), correct rejection rate (*F*_7, 126_ = 18.62, *p* < 0.001; Fig. 3B), and correct option rate (*F*_7, 126_ = 21.62, *p* < 0.001; Fig. 3C) during the NMSS rule-learning phase and the number of sample odours (*F*_3, 54_ = 19.56, *p* < 0.001; Fig. 3D) during the NMMS rule-learning phase, but there was no genotype × training session interaction effect (all *p* > 0.05). These results suggested that 3-month 5 × FAD and WT mice learned the NMSS and NMMS rules well. Analysis of behavioural data from the OWMC test phase revealed a significant effect of genotype on OWMC (*F*_1, 18_ = 24.71, *p* < 0.001; Fig. 3E), averaged errors (*F*_7, 21_ = 27.00, *p* < 0.001; Fig. 3F), and percent correct (*F*_8, 16_ = 12.54, *p* < 0.001; Fig. 3G). We also calculated the percentage of mice that succeeded at each capacity level and found significant differences between the two groups when the number of odours was 6–8 (all *p* < 0.05; Fig. 3H). We have observed that there was no significant difference in weight and daily food consumption between 5 × FAD mice and WT mice (*p* > 0.05; Supplementary Fig. 1), indicating that the OWMC deficit of 5 × FAD was not due to motivation impairment. There was no significant difference in encoding time between two groups at each capacity level (*p* > 0.05; Supplementary Fig. 2a), indicating that the OWMC deficit of 5 × FAD was not due to an abnormality in exploring the sample odours. These results indicated that the memory capacity was impaired in the young adult 5 × FAD mice.

### Brain regions involved in OWMC deficits in 5 × FAD mice

**Fig. 4:**
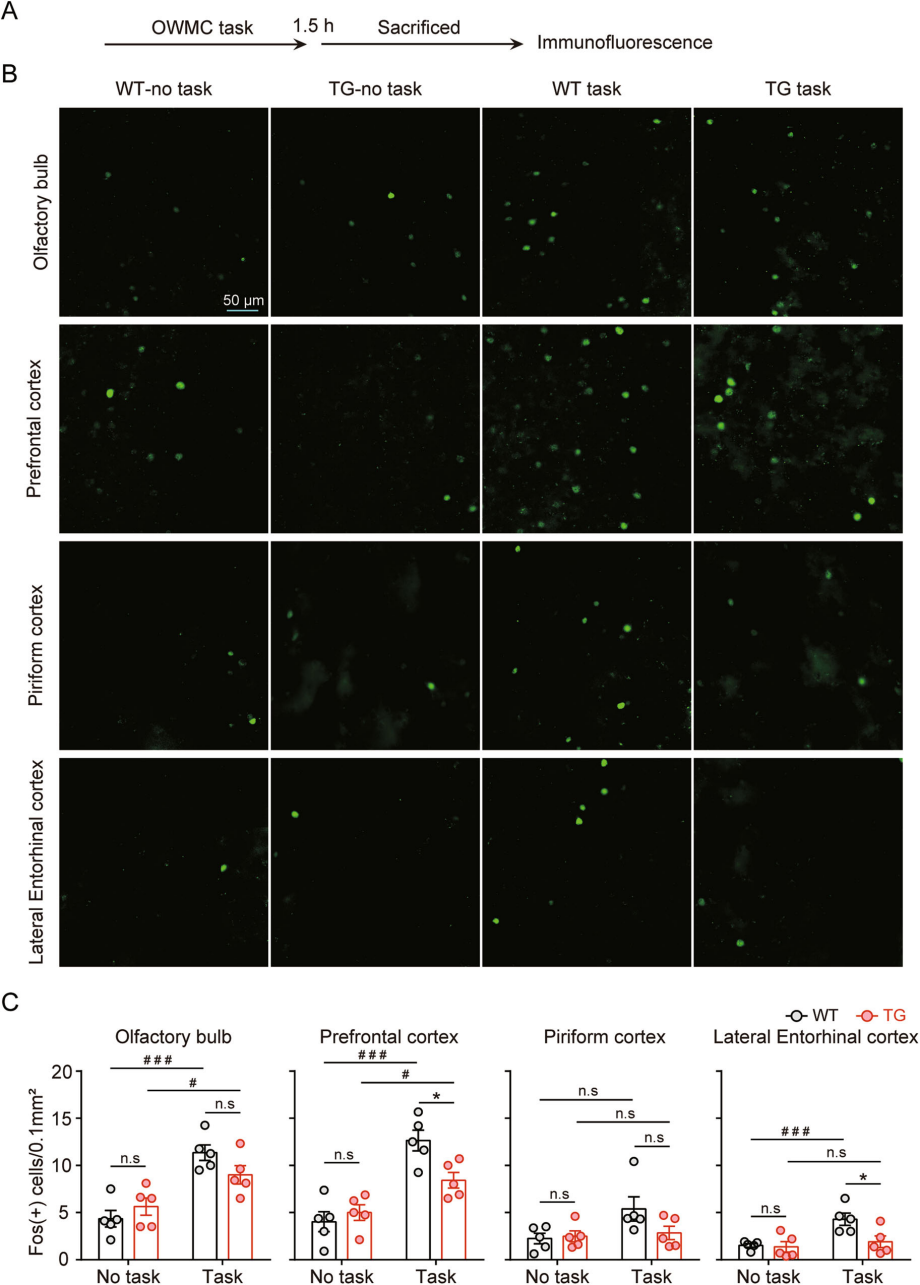
Assessing the neuronal activity of the olfactory bulb, medial prefrontal cortex, piriform cortex and entorhinal cortex induced by the olfactory WM capacity (OWMC) task in 3-month-old 5 × FAD and wild-type (WT) mice. **A** Experimental procedure. **B** Representative images showing Fos protein in the olfactory bulb, medial prefrontal cortex, piriform cortex and entorhinal cortex in different experimental conditions. Scale bars represent 50 μm. **C** Fos protein expression in the olfactory bulb, medial prefrontal cortex, piriform cortex and entorhinal cortex under different experimental conditions. OWMC-induced medial prefrontal cortex and entorhinal cortex activation is reduced in the medial prefrontal cortex of 5 × FAD mice. *n* = 4–5 per experimental condition. Data are the mean ± SEM of the number of Fos. ^#^Compared with the ‘No task’ group, two-way ANOVA, *p* < 0.05. *Compared with the WT group, two-way ANOVA, *p* < 0.05.

We investigated the changes in brain activity induced by OWMC, which could clarify the mechanism of memory capacity deficits in 5 × FAD mice. The mice in Exp. 2 were divided into four groups: WT + no OWMC task, 5 × FAD+ no OWMC task, WT + OWMC task and 5 × FAD + OWMC task. Referring to a previous study^58–61^, two-way ANOVAs, with genotype and OWMC task as the group factors (Fig. 4A), were used to analyse FOS expression in the olfactory cortex, mPFC, piriform cortex and entorhinal cortex, respectively (Fig. 4B), which are considered to be involved in olfactory information processing and WM^62^. The analysis revealed an OWMC task × genotype interaction (Fig. 4C) for the mPFC (*F*_1, 16_ = 7.32, *p* < 0.05) and entorhinal cortex (*F*_1, 16_ = 4.45, *p* = 0.05) and a main effect of the OWMC task in the olfactory bulb (*F*_1, 16_ = 33.04, *p* < 0.001). Either an OWMC task ×genotype interaction or main effect of the OWMC task occurred in the piriform cortex. Post hoc analysis revealed that FOS expression induced by the capacity test was significantly reduced in the mPFC and entorhinal cortex of 5 × FAD mice (*p* < 0.05). We also used the brain regions as one of the between-subject factors for the analysis, and we found a significant effect of genotype (*F*_1, 76_ = 10.36, *p* < 0.01), task (*F*_1, 79_ = 72.52, *p* < 0.001) and brain regions (*F*_4, 79_ = 24.91, *p* < 0.001), as well as the genotype × task interaction (*F*_1, 79_ = 15.65, *p* < 0.001) and task × brain region interaction (*F*_4, 79_ = 4.10, *p* < 0.005); however, there was no significant genotype × brain region interaction (*F*_4, 79_ = 0.55, *p* = 0.70) or genotype × task × brain region interaction (*F*_4, 79_ = 0.33, *p* = 0.86). Our results suggested that the mPFC and entorhinal cortex were likely to reveal the neural mechanism of memory capacity deficits in 5 × FAD mice.

### Deposition of Aß in three-month-old 5 × FAD mice

**Fig. 5:**
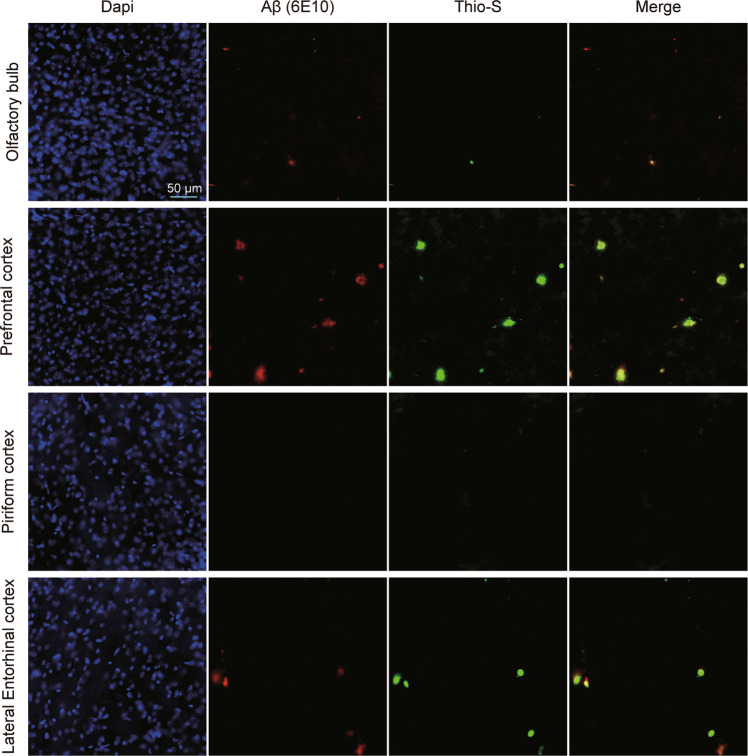
Assessing the amyloid-beta (Aβ) deposits in the olfactory bulb, medial prefrontal cortex, piriform cortex and entorhinal cortex of 5 × FAD mice. **A** Representative images showing Aβ staining in the olfactory bulb, medial prefrontal cortex, piriform cortex and entorhinal cortex in different experimental conditions. Scale bars represent 50 μm.

The deposition of Aβ was observed using a double-staining procedure with thioflavin-S and 6E10 antibodies. Obvious amyloid deposition was found in 3-month-old 5 × FAD mice in the prefrontal cortex, olfactory bulb, piriform cortex and entorhinal cortex (Fig. 5A). The results were in accordance with previous investigations evidencing Aβ expression at the same age in this model^63–65^.

## Discussion

In the present study, we describe a novel olfactory task of WM capacity. We found that OWMC is limited to ~7 in C57BL/6 mice. Then, in 5 × FAD transgenic mice, an animal model of AD, we found that the OWMC of transgenic mice was significantly reduced compared with WT mice, indicating the impairment of WM capacity in 5 × FAD mice. Finally, we found that FOS protein levels were significantly lower in the mPFC and entorhinal cortex but not the olfactory bulb and piriform cortex of 5 × FAD mice after the capacity test, indicating the involvement of the mPFC and entorhinal cortex in the OWMC task, which is also consistent with previous reports showing that FOS expression is increased in the entorhinal cortex and mPFC after the WM task in WT mice^61^. In the present study, we also excluded two possibilities that may affect the interpretation of the assessment of WM capacity. First, the OWMC performance of the mice may be influenced by the odour of the food pellet. However, in the probe trials, we found no differences among baited, unbaited and normal sessions. Second, we excluded the possibility that the mice used spatial cues to select an odour. In each trial, the positions of odours on the choice platform were randomised. In conclusion, we developed a novel mouse procedure of WM capacity, and we also found that lower activation of the mPFC and entorhinal cortex might be involved in impaired OWMC in 5 × FAD mice.

Although capacity is a critical component of WM, few studies have explored capacity limitations using WM tasks in rodents^18^. Inspired by the rodent OST task established by Dudchenko et al.^19^ and Young et al.^21^, we developed a novel paradigm to assess WM capacity in mice. However, there are two differences between the OWMC and OST paradigms. First, in the previous OST design, the sample odours in each trial that subjects remembered comprised the types of odours that were presented in previous trials, so mice may remember certain types of odours repeatedly^19,21,28^. A major design of our paradigm is introducing the NMMS phase, in which the sample odours in each trial are irrelevant to those in previous trials; it requires maintenance of information within a trial, in which not only are subjects required to maintain increasing amounts of information from the odour list, but they are also required to use the information flexibly to make the appropriate response. Consistently, the classic WM capacity test for humans typically requires instant recall of the to-be-remembered items during a single trial^31,32^. To our knowledge, this is the first study to assess the capacity of maintenance of trial-specific information in rodents. Interestingly, we also found that training days (number of trainings) had an improvement effect on the behaviour performance (memory capacity) of the NMMS rule, which may suggest that the NMSS rule is different from the NMMS rule. During NMMS training, the mice learn a strategy switching from the NMSS to the NMMS rule. Second, in the OST paradigm, the animals made few errors and could complete the task with very high accuracy (80% correct or better)^19,22,25,26,66^. A similar phenomenon was also observed in the human version of OST^29,30^. In addition, April et al.^36^ reported that the capacity of well-trained rats to remember odours is not limited to 72 stimuli, although it is not known whether the capacity limits of WM could be defined in mice and humans. To our knowledge, this is the first rodent study to show a limited capacity memory process of the kind proposed in contemporary models of human WM. It is interesting that the OWMC in C57 mice was ~7 in our study, which is similar to the capacity of short-term memory in humans as assessed by Miller^16^, although others have argued that the capacity limit of WM averages ~4 chunks^17^. The performance decrease with increasing duration of the delay period is a typical hallmark of WM paradigms^67,68^. However, like other rodent DNMTS WM tests, there is another explanation for the OWMC paradigm in which rodents could use a mechanism of short-term habituation to complete the memory test. We also examined whether short-term habituation was involved in the mechanisms underlying the OWMC task. We found that compared with the sub-group with no reappearance of an odour in the capacity level 6 test, the sub-group with the reappearance of an odour test did not show significant differences in the correct rate, indicating that short-term habituation is not a significant confound for the OWMC task. However, we cannot thoroughly exclude the possibility that the mice were solving the task based on relative familiarity judgements rather than genuinely solving the task as a human would solve an N-back task. Further studies, such as the DMTS version of OWMC, may help to examine whether habituation/familiarity may underlie the rodents’ performance in this task.

Deficits in WM have a principal role in normal neurocognitive ageing and the rapid cognitive deterioration associated with dementias such as Alzheimer’s disease. WM capacity decreases during old age, which could be due to neurodegeneration^69,70^. Only a few studies have assessed WM capacity in a rodent model of AD^18^. In the current study, we found that the OWMC of transgenic mice was significantly reduced compared with WT mice, indicating the impairment of WM capacity in 5 × FAD mice. It is argued that the deficit in OWMC of 3-month-old 5 × FAD mice is due to deficits in olfactory function. However, the evidence that mice could discriminate odour differences during the NMSS and NMMS phases do not support this possibility. In addition, previous studies have indicated that 6-month-old 5 × FAD mice have no olfactory deficits compared with their WT controls in olfactory detection^71^. Moreover, 2- to 6-month-old 5 × FAD mice showed no deficits on an olfactory maze task, indicating that the 5 × FAD mice were able to detect the odours^72^. Furthermore, it is unlikely that the deficit in OWMC of 3-month-old 5 × FAD was due to a learning deficit in the non-matching rule. Our results demonstrated no significant difference between 5 × FAD and WT mice during the NMSS phase. Interestingly, Shukla et al.^57^ found that 5 × FAD mice showed spontaneous alternation performance at 3 months of age equivalent to that of WT controls, while we observed impairment in the OWMC task. This discrepancy may be attributed to the following reasons. First, spontaneous alternation Y-maze is used to assess spatial WM, while the OWMC is used to detect olfactory WM, and olfactory WM may show impairment at an earlier age compared with spatial WM. Consistently, even at 5 and 8 months of age, the spontaneous alternation of 5 × FAD mice in a Y-maze was not altered in comparison to WT controls^73^. Second, the spontaneous alternation Y-maze is not a difficult task requiring a high cognitive load for rodents; thus, it is not sensitive to detect a moderate impairment of WM in 3-month-old 5 × FAD mice. Consistently, we found that 3-month-old 5 × FAD mice were also able to complete the NMSS and NMMS phases but showed an impairment at a capacity test level of 7 or higher. Finally, the spontaneous alternation test relies on the innate rodent behaviour of alternating unrewarded visits to the arms of the Y-maze. Thus, the continuous alternation version of the Y-maze task is potentially confounded in that if animals always turn left/right in a stereotypical fashion, they can appear to have a high ‘WM’ score, while in the OWMC task, mice must learn a non-match to sample strategy when multiple odours are randomly distributed on the platform. The operant task design may exclude the confounds of stereotypical behaviours. In conclusion, the 5 × FAD mice showed impaired WM capacity in the OWMC paradigm during the early phase of AD. More studies are needed to explore the sex difference and molecular (such as caspase-3 and Aβ) and electrophysiology mechanisms of WM capacity deficit in 5 × FAD mice using the OWMC paradigm.

In the present study, we found that task exposure could increase neuronal activity in all brain regions of interest, including the mPFC, olfactory bulb, entorhinal cortex and piriform cortex, while FOS protein levels were only significantly lower in the mPFC and entorhinal cortex of 5 × FAD mice after the capacity test, indicating that lower activation of the mPFC and entorhinal cortex may be involved in impaired OWMC in 5 × FAD mice. Our results extend previous findings in rodents and non-human primates suggesting that the mPFC regulates the effect of the delay interval on WM^48,74,75^. It is interesting that we did not determine the interaction between genotype × brain regions, although we observed a significant effect of genotype. This result may be explained by the preselected focus on all four brain regions in the present study were of interest. Thus, there was also a similar trend in the olfactory bulb and piriform cortex, although it did not quite reach significance. Mapping the Fos activation in the whole brain could reduce the bias and confirm the specific effects of mPFC and EC on the OWMC task. A human study with hippocampus-damaged patients showed smaller spans for line drawings and colour patterns but only a modest impairment in the OST^29^. In addition, it should be noted that the impact of olfactory deficits should be dissociated from the primary sensory deficits in tasks of odour-related behaviour. However, clinical studies have suggested that olfactory dysfunction is a common and early symptom of many neurodegenerative diseases, particularly AD^76–78^, and olfactory dysfunction in AD mainly presents as an impairment in olfactory identification, which occurs during the early stage of the disease and worsens with the progression of AD, while the olfactory threshold was only present during the late stage of the disease^79^. It should be pointed out that the OWMC task is a kind of olfactory discrimination task, but shows large differences. In the olfactory discrimination task, mice utilise specific odour cues to forage for food, which is appropriate for assessing olfactory discrimination and long-term olfactory memory in mice^80^. However, the OWMC task may be more complex, involving multiple odour discriminations and assessed memory capacity. The olfactory bulb and entorhinal cortex, which are associated with olfactory function, show very early neuropathology in AD^81^. However, we did not find deficits in olfactory function in 3-month-old 5 × FAD mice, and the olfactory bulb and piriform cortex, which were considered to be involved in the olfactory process, showed normal neural activity in 3-month-old 5 × FAD mice after task exposure. Further studies using optogenetic or chemogenetic methods are needed to examine the causal relationship between the mPFC, entorhinal cortex and OWMC, and whether mPFC and entorhinal cortex activity decrease in response to OWMC in AD patients.

WM, as a cognitive process, would require temporal integration of transient stimulus input and hold up to task-relevant stimulus representations^82^. Differences in attention, motivation and motion can act as confounders in the WM assay; in particular, freely moving mice may engage in different types of exploratory foraging. While our results suggest that the impairment observed in the OWMC task was due to a reduced WM capacity, impairments in other cognitive functions such as attention may also have contributions. A similar performance between groups in the NMSS and NMMS training helped to rule out differences in attention and other factors such as motivation and odour discrimination in 5 × FAD mice. However, although we found no differences between WT and 5 × FAD mice in encoding time in the sample zone and correct rate in the choice zone during the NMMS phase, we still do not know whether the different saliencies of odours affect the performance in the capacity test. Calculation of the time animals engaged with each odour sample might help to solve this question. Analysis of the encoding time of each odour can also help to examine the organisation rule of sample encoding and neural correlates of sample encoding. Contextual information may serve as a potent effect factor in cognitive function, and mice may be more susceptible to assign high salience to the alteration of the odour-context association. In the context-odour paired-associate learning task, mice were able to form rapid context-odour associations to gain a food reward^83^. However, this task may not have required knowledge of contextual information for resolution, because the positions of the odours were randomised in each trial; thus, the position information would not aid in finding the reward pellet, but the odour information would help. Classical WM assays are mostly based on visual or auditory modalities, and these types of WM are most frequently studied across different research fields, while olfactory WM has received little scientific attention^84,85^. Although the degree to which WM capacity differs between the visual and auditory modality has been studied, fewer studies have examined the olfactory modality^86–88^. Such modality-specific contributions to WM may be further investigated when visual, auditory and olfactory stimuli are presented. Although research suggests that olfactory function is associated with prodromal AD, to what extent the OWMC paradigm translates to the human situation is unclear, and more studies are needed in both rodent and humans for the detection of prodromal AD. In addition, considering the importance of the delayed period in WM, it will be interesting to explore how OWMC performance changes if the time duration of the waiting zone is extended.

In conclusion, we developed a novel paradigm to assess WM capacity. This task, which can detect early impairment in OWMC related to mPFC dysfunction, is particularly suitable for assessing preclinical therapeutic strategies aiming at the early stage of AD onset.

## Supplementary information

Supplementary information

Figure S1

Figure S2
