# Supplementary material for: A novel paradigm for assessing olfactory working memory capacity in mice
Source: Transl Psychiatry. 2020 Dec 15;10:431. doi: 10.1038/s41398-020-01120-w (PMC7738675; doi:10.1038/s41398-020-01120-w)
Supplement: Supplementary file 1 — Supplementary information [file 41398_2020_1120_MOESM1_ESM.docx]

**Figure S1.** *Supplementary materials.* (**A**) Analysis of body weight of 5×FAD and WT mice at 3 months of age. (**B**) Analysis of daily food consumption of 5×FAD and WT mice at 3 months of age.

**Figure S2.** *Supplementary materials.* (**A**) Sampling time of 5×FAD and WT mice at each capacity level in the capacity test. Data are presented as the mean ± SEM.
