# Supplementary figures and images for: A novel paradigm for assessing olfactory working memory capacity in mice
Source: Transl Psychiatry. 2020 Dec 15;10:431. doi: 10.1038/s41398-020-01120-w (PMC7738675; doi:10.1038/s41398-020-01120-w)

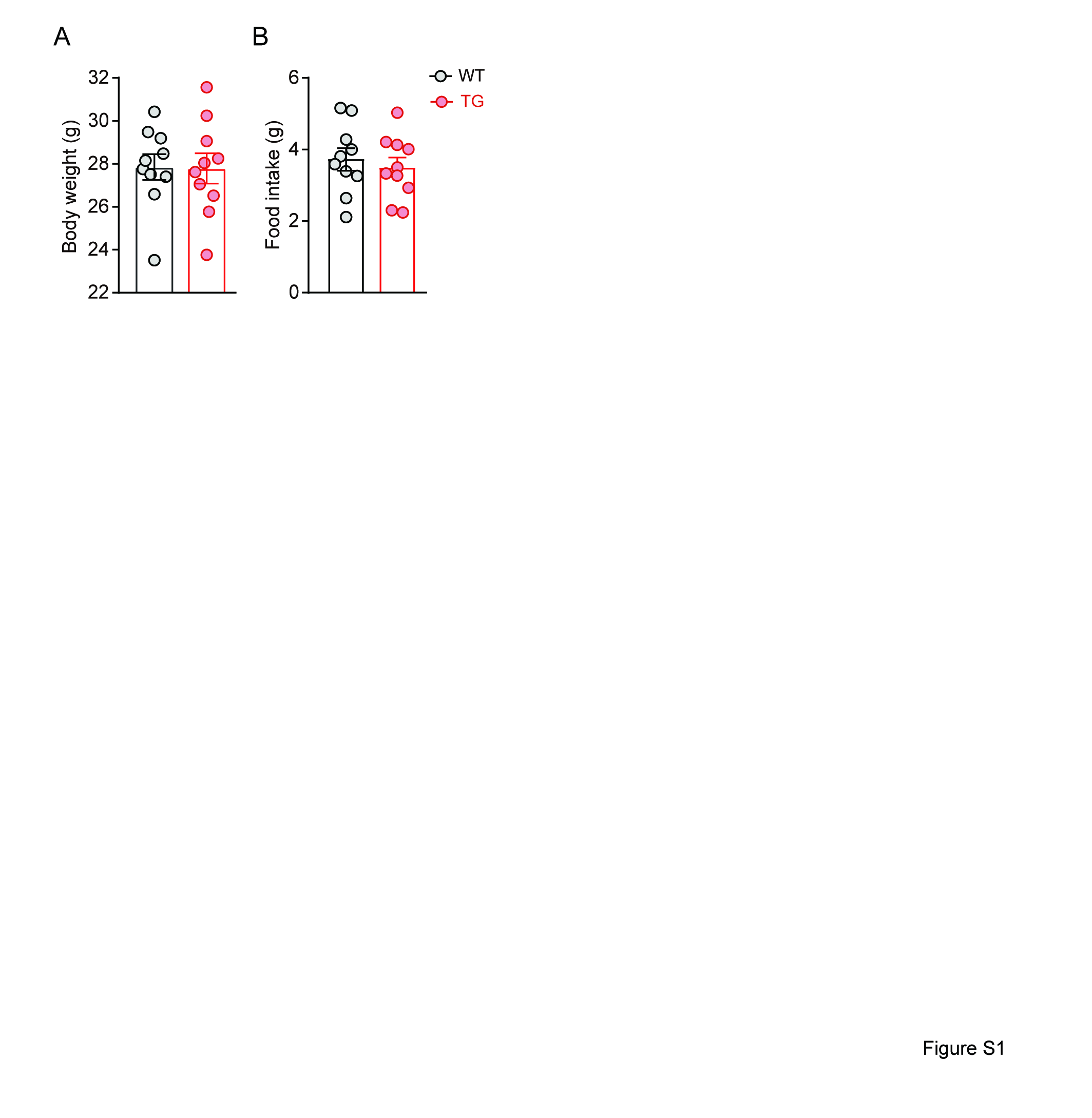

Supplement: Supplementary file 2 — Figure S1 [file 41398_2020_1120_MOESM2_ESM.tif]

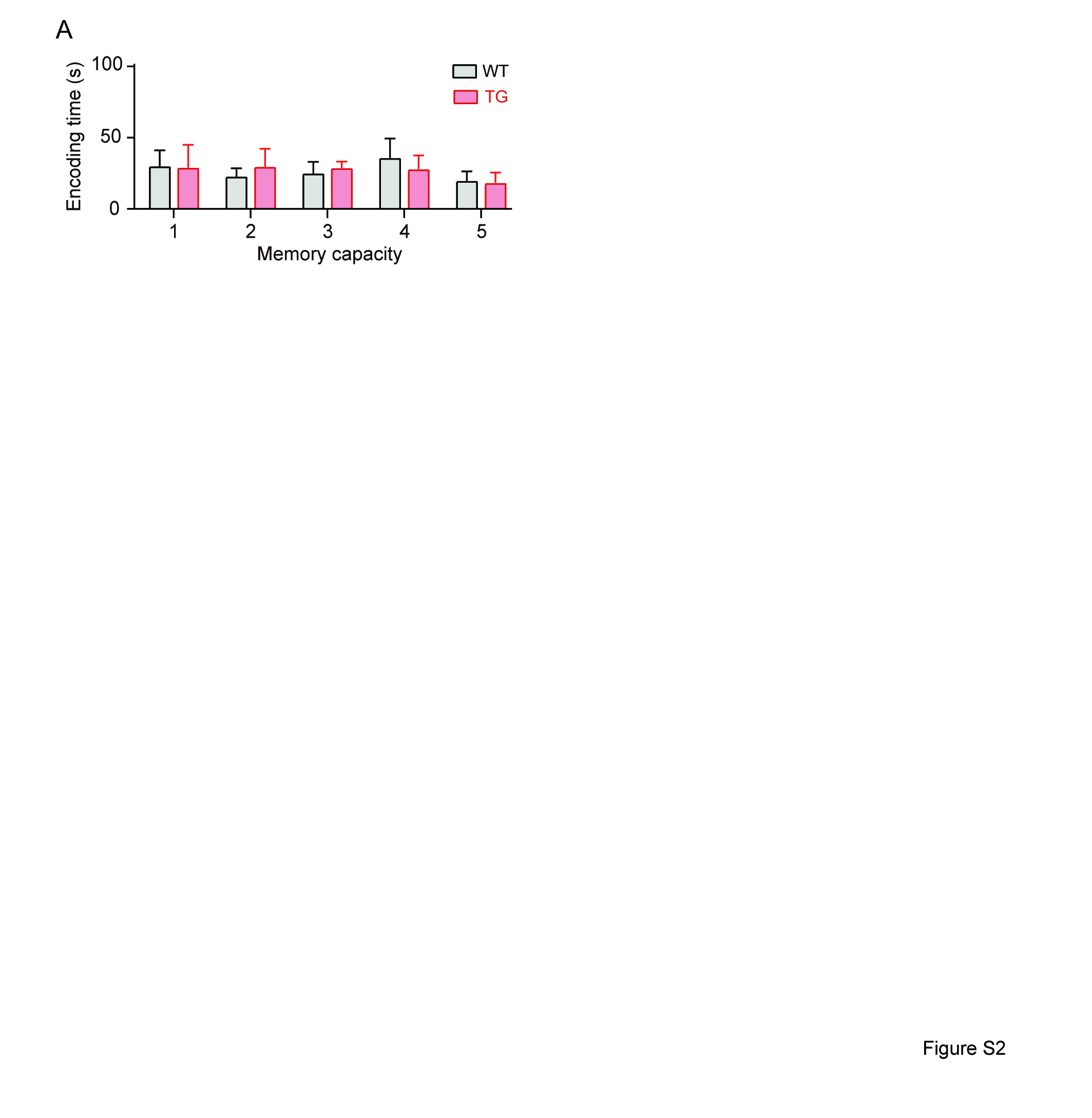

Supplement: Supplementary file 3 — Figure S2 [file 41398_2020_1120_MOESM3_ESM.tif]
